# Small-area and compact CMOS emulator circuit for CMOS/nanoscale memristor co-design

**DOI:** 10.1186/1556-276X-8-454

**Published:** 2013-11-01

**Authors:** SangHak Shin, Jun-Myung Choi, Seongik Cho, Kyeong-Sik Min

**Affiliations:** 1 School of Electrical Engineering, Kookmin University, Seoul 136-702, Korea; 2 Division of Electronics and Information Engineering, Chonbuk National University, Jeonju 561-756, Korea

**Keywords:** Emulator circuit, CMOS emulator circuit, Memristors, Memristive behavior, Nanoscale memristor memory, CMOS/nanoscale memristor co-design, 85.40.-e, 85.35.-p

## Abstract

In this paper, a CMOS emulator circuit that can reproduce nanoscale memristive behavior is proposed. The proposed emulator circuit can mimic the pinched hysteresis loops of nanoscale memristor memory's current-voltage relationship without using any resistor array, complicated circuit blocks, etc. that may occupy very large layout area. Instead of using a resistor array, other complicated circuit blocks, etc., the proposed emulator circuit can describe the nanoscale memristor's current-voltage relationship using a simple voltage-controlled resistor, where its resistance can be programmed by the stored voltage at the state variable capacitor. Comparing the layout area between the previous emulator circuit and the proposed one, the layout area of the proposed emulator circuit is estimated to be 32 times smaller than the previous emulator circuit. The proposed CMOS emulator circuit of nanoscale memristor memory will be very useful in developing hybrid circuits of CMOS/nanoscale memristor memory.

## Background

Memristors are being intensively explored as possible candidate for future memories because of simplicity in fabrication, possibility in three-dimensional integration, compatibility with (complementary metal-oxide-semiconductor) CMOS technology in the fabrication process, and so on. However, real integration of memristors and CMOS circuits is very rarely available to most engineers and scholars who want to be involved in designing various kinds of CMOS circuits using memristors. To help those engineers and scholars who cannot access memristor fabrication technology but want to design memristor circuits, a CMOS emulator circuit that can reproduce the physical hysteresis loop of memristor's voltage-current relationship is needed.

## Methods

Before we develop a CMOS emulator circuit for memristor, memristive behavior should be explained first. The following simple equation (Equation 1) can describe the memristor's current-voltage relationship [1,2]:

(1) $$\begin{array}{c} v\left(t\right)=R_{X}\left(t\right)\cdot i\left(t\right)=\left(R_{\mathrm{SET}}\frac{w\left(t\right)}{D}+R_{\mathrm{RESET}}\left(1-\frac{w\left(t\right)}{D}\right)\right)i\left(t\right)\\ \;=\left(R_{\mathrm{SET}}\frac{q\left(t\right)}{Q_{\mathrm{CRIT}}}+R_{\mathrm{RESET}}\left(1-\frac{q\left(t\right)}{Q_{\mathrm{CRIT}}}\right)\right)i\left(t\right)\;\mathrm{where}\\ \frac{w\left(t\right)}{D}=\mu_{v}\frac{R_{\mathrm{SET}}}{D^{2}}q\left(t\right)=\frac{q\left(t\right)}{Q_{\mathrm{CRIT}}},\;\mathrm{and}\;Q_{\mathrm{CRIT}}=\frac{D^{2}}{\mu_{v}R_{\mathrm{SET}}} \end{array}$$

Here *v*(*t*) and *i*(*t*) represent the voltage and current of memristor, respectively. *R*_*X*_(*t*) is the memristance that changes with respect to time. *R*_SET_ and *R*_RESET_ are SET and RESET resistance, respectively. *w*(*t*) is the effective width of the memristor. *D* is the total drift length of *w*(*t*). *q*(*t*) is an accumulated charge flow through the memristor. *Q*_CRIT_ means an amount of critical charge to RESET-to-SET transition. When *q*(*t*) becomes equal to Q_CRIT_, *R*_*X*_(*t*) is changed to *R*_SET_ from *R*_RESET_. Here *μ*_*v*_ is the mobility of dopant in Equation 1 [1,2].

To describe the memristive behavior that follows the relationship of current and voltage in Equation 1, a few emulator circuits have already been proposed [3-5]. Pershin and Ventra proposed an emulator circuit that is composed of an analog-to-digital converter and micro-controller that are implemented by discrete off-chip devices. Thus, they can be considered too much complicated and too large to be integrated in a single chip [3]. Jung et al. proposed an emulator circuit that is based on CMOS technology [4], where a memristor that should change its resistance in response to the applied current and voltage is implemented by an array of resistors. In the emulator circuit with resistor array, the analog-to-digital converter and the decoder circuit select a proper resistor among many resistors that are placed in the resistor array according to the applied voltage or current [4]. One problem in the emulator circuit [4] is that the voltage-current relationship seems sawtooth. This is because the resolution of memristance change is decided by the resolution of the analog-to-digital converter, as you see in [4]. If we have 4-bit analog-to-digital converter in the emulator circuit, it means that only 16 values of memristance are available. As a result, when we apply a voltage that is a sinusoidal function to the memristor, we can know that its current is increased or decreased like sawtooth. To improve the resolution of memristance change, the resolution of the analog-to-digital converter should be increased too. If the resolution of the analog-to-digital converter is improved from 4 to 5 bit, the voltage-current relationship of the emulator circuit with 5 bit seems to be much finer than the emulator circuit with a 4-bit analog-to-digital converter, as shown in [4]. To improve the resolution twice, however, the number of resistors in the resistor array should be double too. It can cause a large area overhead in realizing this emulator circuit in a single chip. Especially, in implementing memristor array with this emulator circuit, this large area overhead of each memristor emulator cell can be a serious problem because each cell in the memristor array should be realized by this large-area single memristor emulator.

To mitigate the large area overhead of the previous emulator circuit, we propose a new emulator circuit of memristors that is more compact and simpler than the previous emulator circuits [6]. The new emulator circuit does not use a resistor array, an analog-to-digital converter, and so on that usually occupy very large area. Instead of using the complicated circuit blocks that were mentioned just earlier, the new circuit can change its memristance value by a simple voltage-controlled resistor that can be realized by a single n-type metal-oxide-semiconductor field-effect transistor (NMOSFET) device.

### Newly proposed emulator circuit for describing memristive behavior

**Figure 1:**
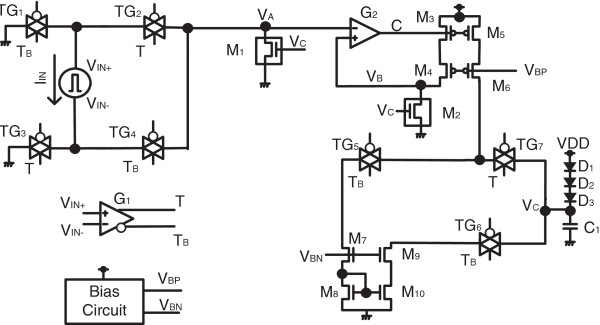
The proposed CMOS emulator circuit for describing memristive behavior.

A schematic of the proposed emulator circuit for describing memristive behavior is shown in Figure 1. The CMOS circuit for emulating memristive behavior is composed of transmission gates, comparators, current mirrors, voltage-controlled resistor, etc. as shown in Figure 1. *V*_IN_ is an input voltage source and *V*_IN+_ and *V*_IN-_represent the anode and cathode of the input voltage source, respectively. In Figure 1, *V*_IN+_ is connected to TG_1_ and TG_2_ that are controlled by T_B_ and T, respectively. Similarly, *V*_IN-_ is connected to TG_3_ and TG_4_ that are controlled by T and T_B_, respectively. When *V*_IN+_ is greater than *V*_IN-_, T becomes high and T_B_ becomes low, by the comparator G_1_. On the contrary, when *V*_IN+_ is smaller than V_IN-_, T becomes low and T_B_ becomes high. Thus, we can know that *V*_IN+_ is connected to *V*_A_ through TG_2_ when *V*_IN+_ is larger than V_IN-_. At the same moment, *V*_IN-_ is connected to the ground potential (GND) by TG_3_. When *V*_IN-_ is larger than *V*_IN+_, *V*_IN-_ is connected to *V*_A_ through TG_4_, and *V*_IN+_ is biased by GND through TG_1_. One thing to note here is that we can deliver the input voltage *V*_IN_ to *V*_A_ without any sacrificial voltage loss, using the transmission gate.

The *V*_IN_ delivering block that is composed of four transmission gates, TG_1_, TG_2_, TG_3_, and TG_4_, can deliver *V*_IN+_ and *V*_IN-_ that are plus and minus polarity of *V*_IN_, respectively, to *V*_A_ that has only plus polarity, not minus. The delivered voltage *V*_A_ is copied exactly to *V*_B_ by the negative feedback circuit that is composed of the OP amp, G_2_, M_3_, and M_4_. Using this circuit block, *V*_B_ can be the same as *V*_A_ by the feedback amplifier with unity gain. *V*_B_ is connected to the voltage-controlled resistor M_2_ that is controlled by *V*_C_. One more thing to note here is that *V*_C_ controls both voltage-controlled resistors M_1_ and M_2_ that are electrically isolated from each other. By doing so, we can separate the memristor's current from the programming current to change the state variable that is stored at the capacitor C_1_. If the memristor's current is not separated from the programming current, the state variable that decides memristance value can be maintained only at the moment when the programming voltage or current is applied to the memristor. If so, the emulator circuit cannot keep its programmed state variable when the applied voltage or current is removed.

*V*_C_ that controls two voltage-controlled resistors M_1_ and M_2_ acts as a state variable in the emulator circuit that is calculated by an amount of stored charge at *C*_1_. When *V*_IN+_ is greater than *V*_IN-_, TG_7_ is on and both TG_5_ and TG_6_ are off. At this time, the current mirror that is composed of M_5_ and M_6_ delivers the programming current to *C*_1_ to increase an amount of stored charge; thereby the state variable becomes larger. On the other hand, when *V*_IN-_ is greater than *V*_IN+_, TG_7_ is off and both TG_5_ and TG_6_ are on. By doing so, we can decrease the amount of charge that is stored at the state variable capacitor*C*_1_. The discharging current path is composed of M_7_, M_8_, M_9_, and M_10_ in Figure 1. Here *V*_BN_ and *V*_BP_ are the biasing voltages for NMOSFETs and PMOSFETs, respectively. *V*_BN_ and *V*_BP_ are made from the biasing circuit that is shown in Figure 1. D_1_, D_2_, and D_3_ are the diodes that are used in the proposed emulator circuit to limit the minimum value of *V*_C_. This minimum value of *V*_C_ is needed to avoid the dead zone which may be caused by the sub-threshold region of the voltage-controlled resistors M_1_ and M_2_. *V*_D_ means the diode voltage of D_1_, D_2_, and D_3_. *V*_DD_ is the power supply voltage of the CMOS emulator circuit in Figure 1.

One more thing to consider here is that the nonlinearity of memristive behaviors can be found when the effective width of memristor, *w*(*t*), in Equation 1 becomes much closer to the boundary constraints [1,7]. This nonlinearity near the boundary values of *w*(*t*) was introduced in the HP model [1] and mathematically modeled by Corinto and Ascoli [7] to describe various nonlinear behaviors of memristors. In terms of implementation, the diode bridge circuit with LCR filter was proposed to reproduce memristive nature with nonlinearity by using a very simple electronic circuit [8]. In this paper, the window function that is used to define two boundary values of the state variable in the HP model [1] is realized in the CMOS emulator circuit that is shown in Figure 1. The emulator circuit in Figure 1 has two boundary values of the state variable that is defined by *V*_C_. Here we can know that the maximum value of *V*_C_ cannot exceed *V*_DD_. And also, *V*_C_ cannot be lower than *V*_DD_-3*V*_D_. Thus, the state variable of *V*_C_ in Figure 1 can exist only between *V*_DD_ and *V*_DD_-3*V*_D_, not being higher than *V*_DD_ and lower than *V*_DD_-3*V*_D_, respectively.

## Results and discussion

**Figure 2:**
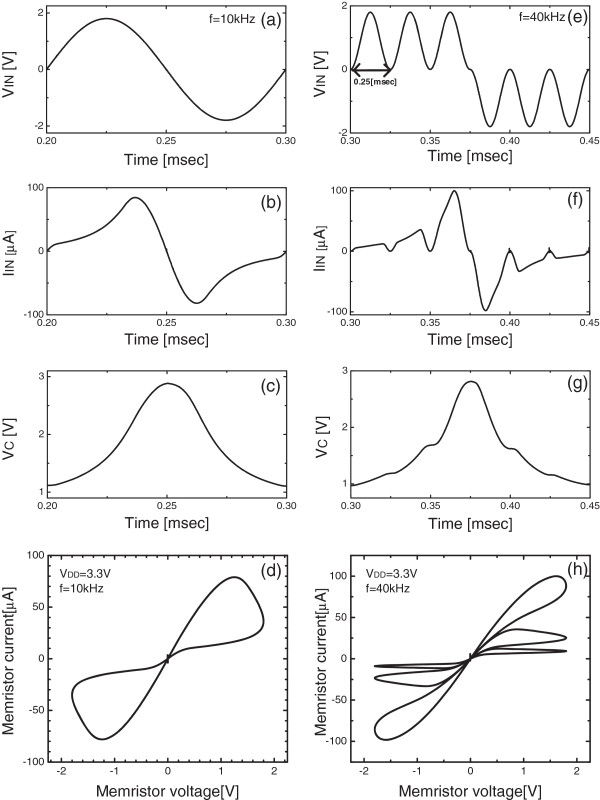
**Simulated voltage waveforms.** The simulated voltage waveforms of **(a)***V*_IN_, **(b)***I*_IN_, **(c)***V*_C_, and **(d)** the pinched hysteresis loop of the voltage-current relationship of the proposed emulator circuit when the sinusoidal frequency is 10 kHz. The simulated voltage waveforms of **(e)***V*_IN_, **(f)***I*_IN_, **(g)***V*_C_, and **(h)** the pinched hysteresis loop of the voltage-current relationship of the proposed emulator circuit when the sinusoidal frequency is 40 kHz.

Figure 2a shows the applied input voltage, *V*_IN_, to the proposed circuit for emulation of memristive behavior. The voltage waveform is sinusoidal and its frequency and magnitude are 10 kHz and 1.8 V, respectively. The memristor's current *I*_IN_ that is emulated by the proposed circuit in Figure 1 is shown in Figure 2b. As the sinusoidal voltage is applied to the emulator circuit in Figure 1, *I*_IN_ changes with respect to time according to the state variable that is represented by *V*_C_, the amount of stored charge at C_1_. When *V*_C_ has the lowest value, it means that the state variable is in RESET state, where the emulator circuit acts like a memristor with RESET resistance. After the half cycle of sinusoidal function, *V*_C_ is charged more and more; thereby *V*_C_ can reach the highest value. With the highest value of V_C_, the state variable can be in SET state, where the emulator circuit can be considered a SET resistance. Figure 2c shows the voltage waveform of *V*_C_ with respect to time. At the starting point of sinusoidal function of *V*_IN_, *V*_C_ is 1.2 V that is decided by D_1_ in Figure 1. After the half cycle of sinusoidal function, *V*_C_ reaches 2.8 V. When one cycle of sinusoidal function is completed, the *V*_C_ value returns to the value at the starting point of sinusoidal function. Figure 2d shows a typical pinched hysteresis loop of a memristor's voltage and current which are emulated by the proposed circuit in Figure 1. In the simulation, *V*_DD_ is 3.3 V and the frequency of sinusoidal function is 10 kHz.

Figure 2e, f, g, h shows the simulation results of the proposed emulator circuit with four times higher frequency of 40 kHz than that of Figure 2a, b, c, d, *V*_IN_, *I*_IN_, *V*_C_, and the pinched hysteresis loop, respectively, with 10 kHz. A sinusoidal voltage with 40 kHz that is applied to the emulator circuit is shown in Figure 2e. Here the first three peaks are for increasing *V*_C_ in Figure 1; thereby, the emulator circuit changes from RESET to SET. The next three peaks are for decreasing the state variable; thus, the emulator circuit can return to RESET. *I*_IN_ and *V*_C_ with the sinusoidal function that is indicated in Figure 2e are shown in Figure 2f, g, respectively. Figure 2h shows the voltage-current relationship of the emulator circuit. In Figure 2h we can see three voltage-current loops at the right and another three voltage-current loops at the left which correspond to the three high peaks and three low peaks in Figure 2e, respectively.

**Figure 3:**
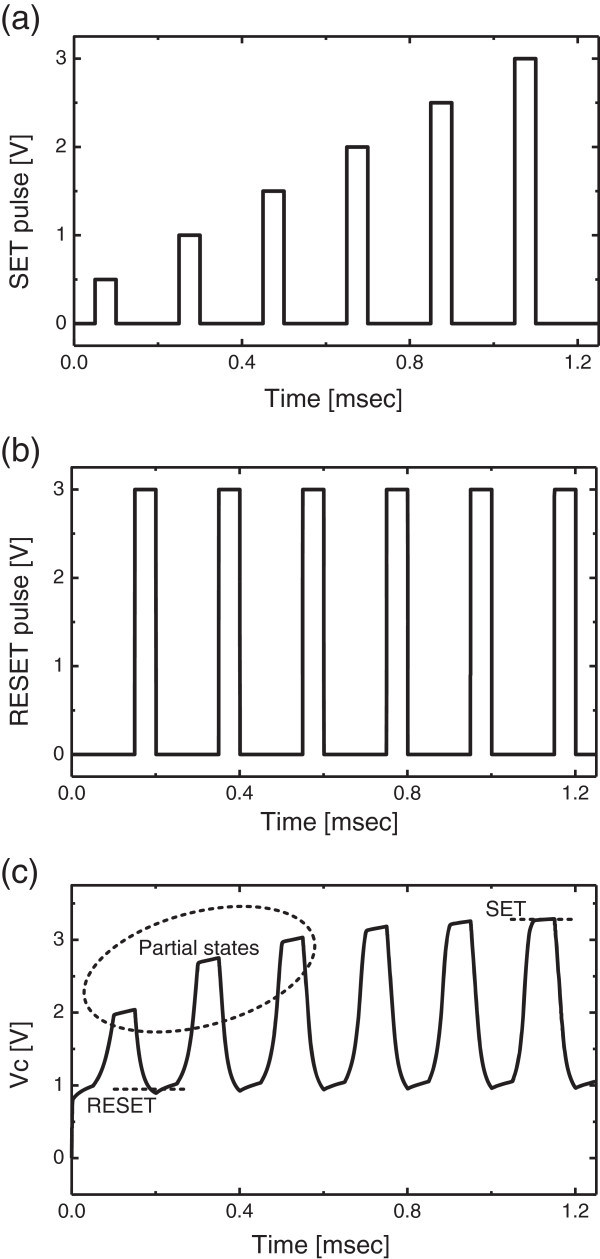
**The simulation results of partial states between 'SET’ state and 'RESET’ state. (a)** The voltage waveform of the SET pulse, **(b)** the voltage waveform of the RESET pulse, and **(c)** the voltage waveform of the state variable that is represented by *V*_C_ in Figure 1.

**Figure 4:**
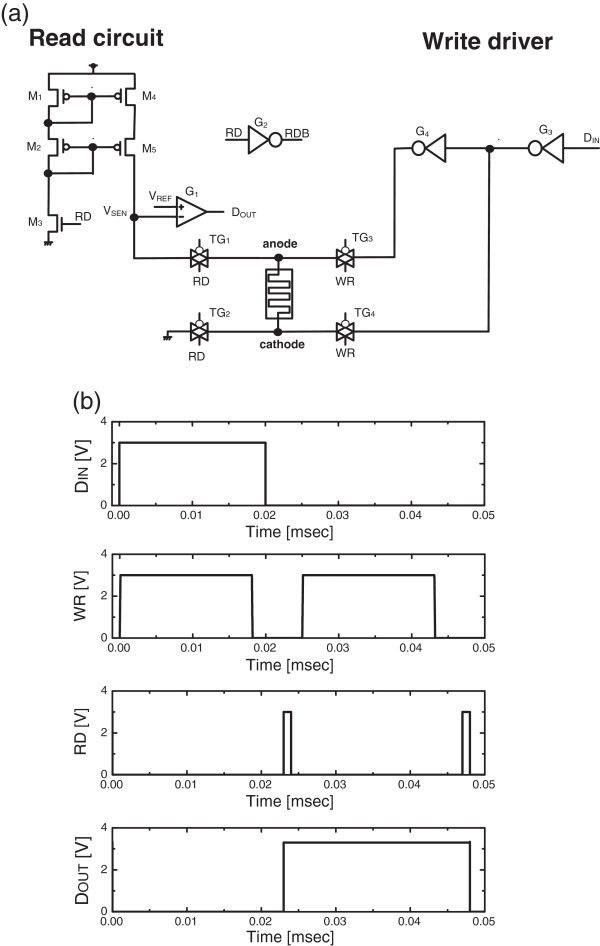
**The read and write circuits for the proposed emulator circuit of memristors and the simulated voltage waveforms. (a)** The read and write circuits for the proposed emulator circuit of memristors. **(b)**The simulated voltage waveforms of *D*_IN_, WR, RD, and *D*_OUT_ that are the input data of the write driver, write command signal, read command signal, and output data of the read circuit, respectively.

Figure 3a shows SET pulses with different amplitude values. Here the amplitude values are increasing monotonically from 0.5 to 3 V. Each SET pulse is followed by a RESET pulse with the fixed amplitude as high as 3 V that is shown in Figure 3b. The state variable that is changed by SET and RESET pulses are shown in Figure 3c. Here *V*_C_ represents the amount of stored charge at C_1_ that controls the voltage-controlled resistor in Figure 1 that acts as memristor. Figure 4a shows the read and write circuits for the proposed emulator circuit of memristors [9,10]. The read circuit is simply composed of a current mirror and comparator. The comparator G_1_ compares the sensing voltage *V*_SEN_ with the reference voltage *V*_REF_. The sensing voltage *V*_SEN_ can change according to the programmed memristance value of the emulator circuit. If the state variable is closer to RESET, the sensing voltage *V*_SEN_ becomes larger due to a large value of memristance. On the contrary, the state variable is in SET, and *V*_SEN_ is smaller than *V*_REF_. Here *D*_OUT_ is the output voltage of the read circuit. G_2_ is the inverter for RD that is the 'read’ command signal. TG_1_ and TG_2_ are the transmission gates for the read operation. When RD is high, TG_1_ and TG_2_ are on. On the contrary, TG_3_ and TG_4_ are on for the 'write’ operation that is activated by the write command signal WR. The input data *D*_IN_ drives the inverter G_3_. And G_3_ drives the next inverter G_4_. The anode and cathode of the proposed emulator circuit are driven by the two inverters, G_3_ and G_4_, respectively. Figure 4b shows the voltage waveforms of *D*_IN_, WR, RD, and *D*_OUT_.

**Figure 5:**
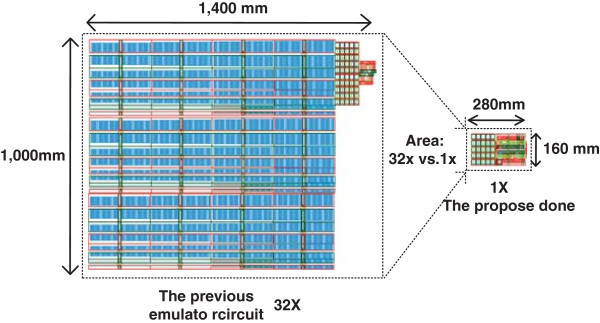
**Comparison of layout area between the previous emulator circuit [**[4]**] and the proposed emulator circuit.** The previous emulator circuit has a layout area as large as 1,400 × 1,000 μm^2^and the proposed emulator can be placed in an area as small as 280 × 160 μm^2^.

Figure 5 compares the layout area of the previous emulator circuit [4] and the proposed emulator circuit. Because the resistor array is not used in the proposed circuit and the analog-to-digital converter and decoder are eliminated in this paper, the layout area of the previous emulator circuit is estimated to be 32 times larger than the emulator circuit proposed in this paper. The design rule used in this layout is MagnaChip 0.35-μm technology.

## Conclusions

In this paper, a CMOS circuit that could emulate memristive behavior was proposed. The proposed emulator circuit could mimic the pinched hysteresis loops of a memristor's current-voltage relationship without using a resistor array and complicated circuit blocks that may occupy very large layout area. Instead of using a resistor array, other complicated circuit blocks, etc., the proposed emulator circuit could mimic memristive behavior using simple voltage-controlled resistors, where the resistance can be programmed by the stored voltage at the state variable capacitor. Comparing the layout area between the previous emulator circuit and the proposed one, the layout area of the emulator circuit proposed in this paper was estimated to be 32 times smaller than the previous emulator circuit.

## Competing interests

The authors declare that they have no competing interests.

## Authors’ contributions

All authors have contributed to the submitted manuscript of the present work. KSM defined the research topic. SHS and JMC did the simulation and layout. SC provided critical comments on the draft manuscript. KSM wrote the paper. All authors read and approved the final manuscript.

## Authors’ information

SHS and JMC are M.S. students who are studying at the School of Electrical Engineering, Kookmin University, Seoul, Korea. SC is a professor at the Division of Electronics and Information Engineering, Chonbuk National University, Jeonju, Korea. KSM is a professor at the School of Electrical Engineering, Kookmin University, Seoul, Korea.
